# Notes of the Week

**Published:** 1921-09-10

**Authors:** 


### NOTES OF THE WEEK.

### PRINCE HENRY AND HEXHAM.

It is announced that Prince Henry will visit Hexham on September 29 to open the War Memorial Hospital and to unveil a war memorial in the Abbey grounds.

### THE PRINCE AS LANDLORD.

An excellent example, which many might follow according to their ability, has been set by H.R.H. the Prince of Wales in giving generous assistance towards solving the housing problem in Lambeth. The assistance comes from the Duchy of Cornwall and goes to help the work of reconstructing old houses, clearing slum areas, and similar problems. The Medical Officer of Health, Dr. Priestley, in his recent report, explains the essential contrast between the slipshod methods by which so much of this landlord's work is often done and the very thorough manner, the best materials, and the supervised labour which are being devoted to the Prince's property. Much more work is being done than the Council's housing inspectors have asked for in their schedules!

### BARRISTER, SURGEON, MAGISTRATE.

By succeeding Mr. Bros as magistrate at Clerkenwell Mr. Samuel Fleming has, we should imagine, set up something of a record, for, in addition to the above, he is a barrister and a qualified surgeon. Mr. Fleming, who is a Master of Surgery and has been Recorder of Doncaster since 1920, rendered valuable service during the war, becoming at the outbreak of hostilities a judge advocate, and later holding the positions of legal adviser to the Army Medical Department and Deputy Assistant Director-General.

### HOSPITAL TENNIS.

The victory of the London Hospital last week over Guy's in the final round for the Inter-Hospital Lawn Tennis Challenge Cup gives the cup to the London for the second year in succession. A large gathering watched the game in the grounds of St. Marylebone Infirmary. Thirty-two hospitals in and near London entered for the competition, which is a public illustration of the popularity of tennis among hospital staffs and perhaps especially nurses.

### INFANT MORTALITY IN SUMMER.

In keeping with records of previous years, London infantile mortality advanced considerably during July and August. Deaths from diarrhœa and enteritis alone gave an average rate of eleven per thousand, and the weekly numbers steadily increased throughout the period mentioned. It has been so frequently urged that these cases are to a great extent preventable that it is useless again to stress the point, but there is ample indication of the good that welfare work and centres may do, when we note that in one week in London 154 young babies died from this one cause.

### VENEREAL DISEASE.

Without adopting the view, expressed in certain quarters, that the Ministry of Health is utterly and entirely wrong in its handling of the venereal diseases problem, yet we think there seems little doubt that the time has come to give prevention a better chance. Sir James Crichton-Browne put the case clearly and strongly in his recent address at Bath, and we shall hope soon to see the matter dealt with on more logical and practical lines. There is one question which before everything else must be finally settled. Compromises and half-measures are useless. Are we or are we not to make known to the public the efficient means which we now possess of preventing venereal disease?

### THE LONDON "TOLL" EXCHANGE.

Owing to the increase of traffic passing over the trunk telephone system to and from London, a new exchange to handle messages to towns within a twenty-five miles radius of London has been constituted and is now approaching completion. It will be known as the London Toll Exchange and is expected to be in full operation by the end of September. In the process of designing the switching apparatus opportunity has been taken to introduce new facilities for the operation of the calls, so that connections to the provincial towns which are served by the exchange may be obtained on demand. If towns within the radius are being called the request must be for "Toll" instead of "Trunks." It is hoped that in the majority of cases the call will be completed whilst the caller waits on the line, as in the local service.

### EPILEPSY.

The matter of using salt solution of certain concentration as a means for reducing the pressure inside the skull is being worked out by the Harvard Medical School Laboratory. The use of salt solution, intravenously, in altering the intra-cranial pressure is not entirely new, but the fact that similar effects may possibly be obtained simply by swallowing salines is distinctly interesting. This reduction of pressure would mean that protrusion of brain substance into bony injuries of the skull might be prevented or delayed. Also, in the case of epilepsy, where the fit is preceded by a rise of pressure, a means of prevention may be at hand. The method undoubtedly requires further investigation, but even at its present stage has decided possibility.

### MASSAGE BY THE BLIND.

The second annual report of the Association of Certificated Blind Masseurs, in connection with the National Institute for the Blind, indicates the increasing utility of this organisation, the mission of which is to promote the interests of blind persons who have been trained in the useful art of massage. The membership has increased to 146; eighty-eight of these are blind soldier masseurs, thirty civilian masseurs, and twenty-eight masseuses.

### THE SURPLUS OF THE APPROVED SOCIETIES.

The reasonable demand that the hospitals should receive a share of the seven million surplus accumulated by the approved societies associated with the National Health Insurance Act of 1911, has as yet achieved little result. Isolated negotiations have been carried on with varying and, on the whole, disappointing results. Perhaps, therefore, there is something promising in the suggestion put forward by *Progress* that a grant of at least £500,000 should be made to the voluntary hospitals from the surplus of the approved societies, if only that the million recommended by the Cave Committee, of which the Government has promised but half, may be found. The Government grant is really a recompense, however inadequate, for services rendered; since at least an equal debt is owing from the approved societies, now is the time to press for payment.

### ECONOMY IN WHITEHALL.

A circular recently issued by the Ministry of Health to the Poor-Law authorities will conduce to economy both at the central office and in local centres. Hitherto every fresh appointment, no matter how subordinate, has been submitted to the Minister of Health for his approval. This approval was merely a formal matter in most instances, but it involved both delay and an unnecessary amount of clerical work. It is proposed that in future an annual statement of local needs should be issued, including the proposals for the coming year, and that, if this is approved, individual cases should be left to the discretion of the local Guardians. In minor matters of finance also, such as the purchasing of goods for an institution or the carrying out of works, it is proposed to allow more freedom to the local people. This change will mean a great saving in stationery alone, and should enable both the central and local offices to reduce their staff, besides putting an end to much otherwise inevitable delay in arrangements. Such a simplification of procedure is wholly commendable.

### THE SYLLABUS OF THE GENERAL NURSING COUNCIL.

The small hospitals are complaining that the Syllabus of the General Nursing Council would be difficult for them to carry out because it is overloaded. These opinions have been communicated to the Rev. G. B. Cronshaw, who represents the British Hospitals Association upon the General Nursing Council. We hope that some solution of these difficulties will be found. At all events the position of the smaller hospitals and the requirements of the Council could not be considered by a more capable person. That the British Hospitals Association has been able to voice the smaller hospitals in this matter is an indication of the services which it can render, and should encourage all not yet affiliated to become members.

### HOSPITAL STAFFS AND UNEMPLOYMENT INSURANCE.

The Council of the British Hospitals Association has taken the opinion of Sir John Paget, Bart., K.C., on the advisability of an appeal against the decision of the Minister in regard to the position of sisters and nurses under the Unemployment Insurance Act of 1920, Section 10. Counsel's opinion is that the only grounds of appeal under the Act offer no hope of success. In his view it would be vain to appeal on the ground that the employment of sisters and nurses is employment in domestic service within the meaning of paragraph (*b*) of Part II. of the First Schedule of the Act, in which case they would be excepted under the paragraph unless the employment is in any trade or business carried on for the purposes of gain. He holds the same opinion of the ground that a hospital comes under the definition of a "Local or Public Authority," the employees of which are excluded under certain conditions (see Leaflet U.I.A. 479, "Certificates of Exemption"). There are no other grounds of appeal.

### "THE MISSION HOSPITAL."

On the whole we think that the medical committee of the Church Missionary Society has done well to decide, after much deliberation, to change the title of its monthly organ—*Mercy and Truth*: a Record of Medical Missions—to *The Mission Hospital*. The familiar name did strictly cover the two fields of the Society's work—healing and preaching, but in the course of time the name has come to suggest mission work solely, and no uninformed person interested in hospitals would expect to find institutional news in a paper with such a title. We ourselves are particularly glad of the alteration, because we have for years pleaded that the medical side of mission work should be better known, and that the doings of these remote and interesting hospitals should not remain shrouded in obscurity. The change of name is likely to attract further interest to these institutions, and the September number of the magazine is remarkable for the amount of hospital and medical matters that it contains. We understand that each month the rechristened magazine will have a frontispiece representing one of the C.M.S. mission hospitals, followed by a leading article upon that institution's work. If we might venture a question of our own it would be, Is not the present narrow shape of the magazine open to improvement? Hitherto it has been as long and as narrow as a glove. Now that the title is being changed, it might be suggested that more harmonious proportions of length and breadth would make the journal look more interesting than it does at present Too many circulars are of this shape to make it inviting.

### WARRINGTON'S NEW SANATORIUM.

Because of a shortage of beds at Sankey Sanatorium, the Warrington Corporation has acquired and converted Hefferston Grange, Weaverham, which has now been formally opened. The pavilio[illegible] near the main building already accommodates thirty patients. Under a resident medical officer the staff will consist of a matron, a night superintendent, eight nurses, and fourteen maids. The Sankey Sanatorium provides for twenty-six patients, but the number of beds at which the Corporation aims is eighty. The resident medical officer will assist the M.O.H. in the work of the borough.

